# Community-engaged and community-based participatory research to promote American Heart Association Life’s Simple 7 among African American adults: A systematic review

**DOI:** 10.1371/journal.pone.0238374

**Published:** 2020-09-01

**Authors:** Rana Elgazzar, Timiya S. Nolan, Joshua J. Joseph, Emmanuela B. Aboagye-Mensah, Rosevine A. Azap, Darrell M. Gray

**Affiliations:** 1 The Ohio State University College of Medicine, Columbus, OH, United States of America; 2 The Ohio State University College of Nursing, Columbus, OH, United States of America; 3 The Ohio State University Comprehensive Cancer Center, Columbus, OH, United States of America; San Diego State University, UNITED STATES

## Abstract

**Background:**

Cardiovascular disease (CVD) is the leading cause of death in the United States and African Americans (AA) have a disproportionately greater burden of CVD as compared to Whites. The American Heart Association (AHA) Life’s Simple 7 (LS7) framework outlines goals for attaining ideal cardiovascular health. Yet, there is a lack of evidence summarizing best practices to maximize LS7 attainment. The objective of the present study was to systematically review the extant peer-reviewed literature on community-engaged and community-based participatory research (CBPR) aimed at improving one or more LS7 metrics among AA.

**Methods:**

PubMed, CINAHL, and Embase databases were searched. We included articles that reported quantitative results for one or more of the following LS7 metrics: physical activity, diet, cholesterol, blood pressure, body mass index, smoking, and glycemia. We included analyses with a greater than 50% AA study population focused on adults (≥18 years of age).

**Results:**

Of the 1008 unique studies identified, 54 met inclusion criteria; 27 of which were randomized controlled trials. 50% of studies assessed more than one LS7 metric but only two studies evaluated all seven of the LS7 metrics. No studies had a high proportion of AA males. 40 studies improved at least one LS7 metric at the study end-point. Formative research was used in many studies to guide intervention design. Studies were of varying quality, but overall rated “fair” using a modified approach to the National Institute of Health quality assessment tool.

**Conclusion:**

There is insufficient data to recommend a specific community-engaged or CBPR intervention to improve attainment of LS7 metrics among AA. Future studies using rigorous methodology with increased gender diversity and utilizing the AHA LS7 framework are required to establish a validated program to improve LS7 in AAs.

## Introduction

Cardiovascular disease (CVD) is the leading cause of death in the United States [1]. CVD incidence and mortality is highest among racial and ethnic minorities [2]. This is of critical importance as the percentage of ethnic minorities are projected to vastly increase in the United States. Estimates show that African Americans (AA) will see a 42% population increase between 2014 and 2060 [3]. AA, in particular, have a disproportionately greater burden of CVD and lower life expectancy, as compared to Whites [1, 4, 5]. CVD disparities among AA are facilitated by a high prevalence of a multitude of risk factors, such as obesity, hypertension, and diabetes mellitus [6], in addition to the interaction of social and behavioral health factors [7]. Furthermore, there are striking gender differences which interface racial health disparities. The burden of chronic disease is substantially higher in AA males, who have the highest age-adjusted all-cause mortality of any race-gender group in the U.S [8]. In 2015, the life expectancy of non-Hispanic Black males was 71.8 years compared to 78.1 for Black females and 78.7 for Whites of both sexes [8]. In addition to the higher rates of CVD, Black men are also twice as likely to die from CVD than White men [9].

Given that behavioral and biological factors contribute to a large percentage of health, the American Heart Association (AHA) in 2010 outlined goals for reaching ideal cardiovascular health through the Life’s Simple 7 (LS7) framework [10]. The framework consists of modifiable lifestyle behaviors and biometric factors, including smoking status, body mass index (BMI), physical activity, diet, total cholesterol, blood pressure, and blood glucose. There have been numerous studies showing associations of higher attainment of LS7 with lower risk of CVD, diabetes, and cancer. Additionally, interventions addressing multiple risk factors are more likely to reduce rates of fatal CVD when compared to single risk factor interventions [11]. However, little evidence exists reviewing best practices to maximize LS7 attainment in communities and populations that have high prevalence of CVD along with various chronic diseases and all-cause mortality.

Multi-ethnic cohorts including AA [12–14] and studies focused specifically on AAs [13] showed that higher attainment of ideal cardiovascular health as defined by the seven factors (i.e., smoking abstinence or cessation, BMI <25 kg/m^2^, adequate levels of physical activity, healthy diet, total cholesterol <200 mg/dL, blood pressure <120/80 mmHg, and fasting glucose <100 mg/dL in the absence of diabetes mellitus) is associated with significantly lower risk of CVD [10]. Moreover, ideal cardiovascular health is associated with lower risk of incident cancer and type 2 diabetes [15, 16]. Compared to Whites, Chinese Americans and Hispanic Americans, AAs have lower attainment of ideal LS7 metrics [17]. In addition to individual lifestyle and behavior factors, there are environmental and psychosocial factors that are framed within the social determinants of health (SDOH) that impact the disproportionate burden of chronic disease in AAs. The SDOH are “the conditions in which people are born, grow, live, work and age,” which include domains such as economic stability, education, and built environment [18]. In general, AAs are disproportionately burdened by poor status of SDOH [19]. These upstream factors play a key role in the development of CVD risk factors [20]. Racism is an additional burden on AAs that may impact the individual (individual racism) and underlies inequities in the social determinants of health across AA communities through structural racism, such as lower educational levels, higher poverty, higher violence, and exposure to environmental toxins [21, 22]. On the individual level, there is evidence that racism contributes directly to the higher incidence of HTN in AA [23] and interpersonal racism is associated with increased long-term stress levels [24]. This is further magnified by the social and environmental barriers that stem from institutional racism, such as its negative effect on socioeconomic status and healthcare access, highlighted by the impact of redlining [25, 26].

Successful efforts to activate and engage large numbers of AAs to attain ideal cardiovascular health with the goal of reducing disparities in CVD, diabetes, and cancer are vastly needed. However, successfully implementing such interventions can be challenging. Community-engaged research can aid in mitigating this challenge. It overcomes some limitations of the traditional medical model by integrating the cultural, social, and environmental contexts that underlie clinical and public health initiatives [27]. Guided by the social ecological model, community-engaged research is broadly characterized as the process of multidisciplinary collaborative work among community groups sharing special interests to address problems and customize solutions specific to that community [28]. Community-based participatory research (CBPR) is one type of community-engaged research.

CBPR is a highly involved cooperative approach which mobilizes and empowers communities through research partnerships to develop, implement, test, and sustain effective interventions targeted toward a population’s unique goals and challenges [29]. While community-engaged research gives communities shared ownership over the products of research, CBPR goes a step further by necessitating that community stakeholders participate in all stages of research [29]. Through the shared exchange of expertise, CBPR focuses on improving health by involving community members, public leaders, civic organizations, and academic institutions at all levels of research development and translation. CBPR, as well as community engaged research overall, has demonstrated favorable intervention acceptability [30]. Furthermore, utilization of such strategies may be critical to engaging and reducing disparities in attainment of ideal LS7 metrics among AAs.

Within the community-engaged and CBPR frameworks, formative research is often utilized in order to culturally tailor interventions to the population of interest, especially when targeting lifestyle changes [31]. Rather than testing a specific hypothesis, formative research uses qualitative and/or quantitative methods such as interviews, focus groups, direct observation, and surveys, to inform the design and implementation of interventions which take into account community attitudes, needs, and barriers [32].

With AAs attaining fewer ideal components of the LS7 framework, improved knowledge of effective disease prevention strategies aiming to mitigate the disproportionate rates of premature morbidity and mortality among AA due to CVD, diabetes, and cancer are necessary. LS7 interventions developed using CBPR and community-engaged research principles may have significant impact at the individual and population levels. However, best practices to conduct such work have yet to be established. This systematic review aims to examine the current literature on interventions targeting LS7 improvement in AAs. Specifically, this review will focus on CBPR and community-engaged research used to evaluate efficacy and effectiveness of interventions using one or more LS7 metrics in AAs.

## Methods

We conducted a systematic review of English-language literature to identify and synthesize extant literature on the assessment of one or more LS7 metrics within CBPR and community-engaged interventions for AAs. We searched MESH/Headings and keywords for terms associated with *African American*, *black*, *community-based participatory research*, *community engagement*, *cardiovascular health*, *cardiovascular disease*, *physical activity*, *exercise*, *healthy diet*, *nutrition*, *smoking*, *smoking cessation*, *body weight*, *BMI*, *blood pressure*, *hypertension*, *blood sugar*, *diabetes*, *glycemic control*, *cholesterol*, *and cholesterol VLDL*. In June 2018, we queried PubMed, Embase, and CINAHL databases for relevant articles without time constraints. The search was repeated in December 2019 to include new articles published up to December 16^th^, 2019. Search strategies are presented in the S1 Appendix. All articles were imported into Covidence Software for reference management [33]. There is not a registered protocol associated with this systematic review.

Articles were screened and reviewed based on selection criteria. Inclusion criteria were applied to meet the study purpose. We included articles that reported quantitative results for the outcomes of interest relating to one or more of the following LS7 health components: physical activity, diet (such as fruit and vegetable consumption), cholesterol, blood pressure, body mass index, smoking, and glycemia (glucose or hemoglobin A1c). Interventional study designs were eligible for inclusion. These included both experimental designs such as randomized controlled trials and cluster randomized trials, as well as quasi-experimental designs such as variations of pre-post tests with or without a comparison group. We included analyses with a greater than 50% AA study population focused on adults (≥18 years of age). Interventions included in the review employed principles of community-engaged research (at a minimum) or community-based participatory research. For example, interventions involving recruited participants from the target population, carried out in community settings such as churches, schools, and businesses, as well as those involving community coalitions in the planning, conduct, and/or analysis of the research study were included. Articles were excluded if they did not meet inclusion criteria. Specifically, studies were excluded if they did not involve ≥ 50% AA adults, present quantitative results for the outcomes of interest, or use community-engaged or CBPR methods.

The following describes the iterative process of review. Citations were gathered through the literature search. There were 124 duplicates which were subsequently removed. Four reviewers (RE, TSN, JJJ, and DGII) reviewed 1008 titles and abstracts for inclusion and exclusion. Disagreements were resolved through review and consensus by all four reviewers. Full text review of 226 articles was conducted in the same manner based on inclusion and exclusion criteria. During full text review, we used ascendency and descendancy to identify relevant articles for inclusion. After full text review, two reviewers (RE, TSN, JJJ, and DGII) performed the critical appraisal (quality assessment) of each of the 54 remaining articles using the National Heart, Lung, and Blood Institute Study Quality Assessment Tools from the National Institute of Health (NIH) with a modified approach [34]. This NIH tool evaluates internal and external validity, in addition to sources of bias, confounding, and other potential flaws specific to each study design. For example, criteria being assessed for controlled studies included methods of randomization, blinding, and sample size. By affirming or negating each query in the tool, studies can be assessed for an overall quality rating of “good”, “fair”, or “poor.” Our team determined “good” studies to be those affirming at least 70% of the items in the assessment tool. “Fair” studies affirmed 50%-69% and “poor” studies affirmed less than 50% of the items in the tool. No studies were excluded based on the quality rating. Questions comprising the quality assessment tool for each study design are included in the S1 Appendix. Finally, data was extracted from selected articles and collated into matrices for content analysis. Data points include author, study design, description of sample, mean age of participants, LS7 metric(s) assessed, description of intervention, and results.

## Results

### Study selection

**Fig 1:**
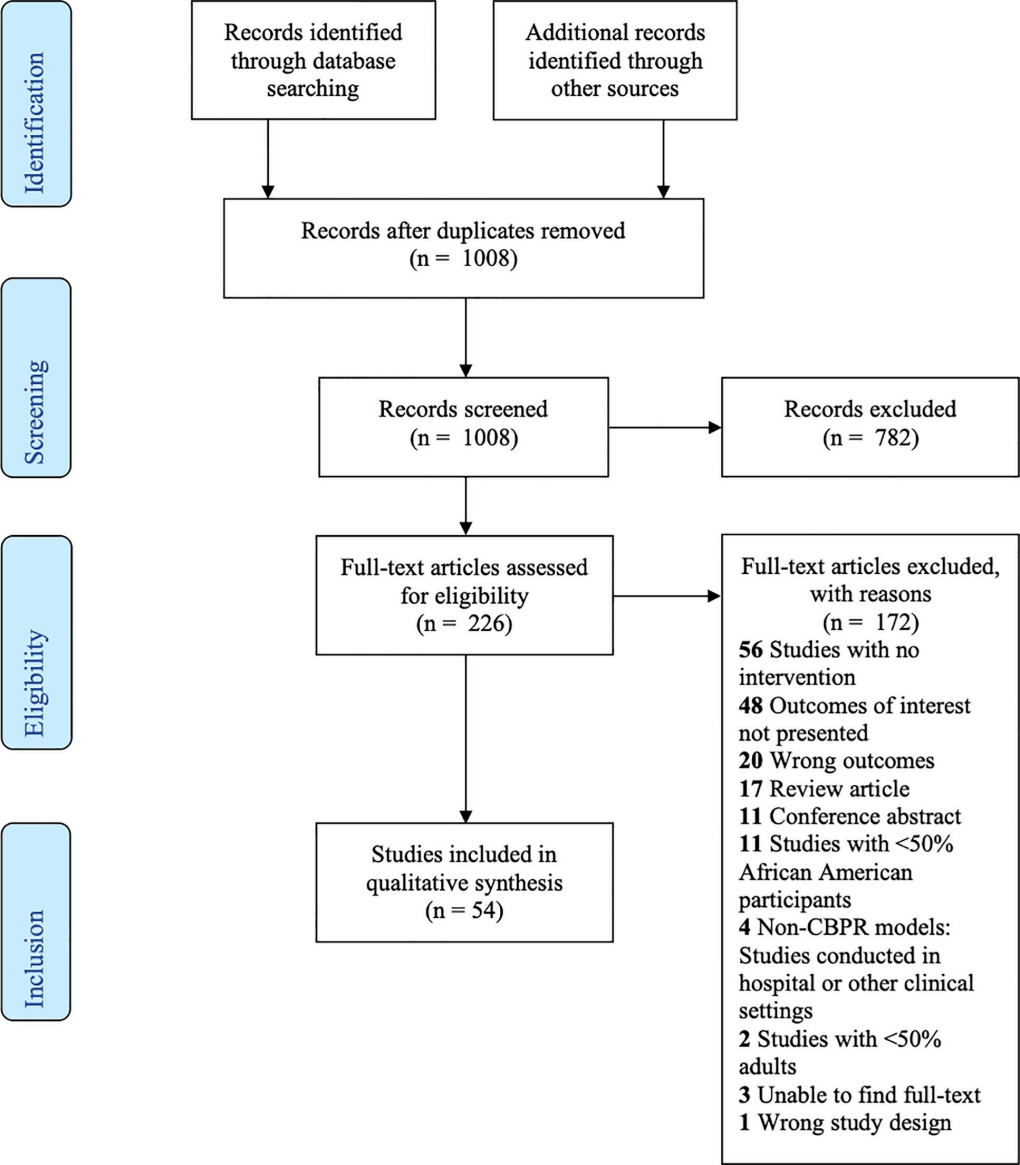
PRISMA flow diagram.

A total of 966 citations were gathered through the initial literature search in Pubmed, CINAHL, and Embase. An additional 12 studies were gathered through other sources (reference searches). Using the same search strategies, 154 citations were added in December 2019. In total, 1008 studies remained after 124 duplicate articles were removed. We excluded 782 citations which were irrelevant based on review of titles and abstracts. We collected full text articles for the remaining 226 studies which were reviewed based on inclusion and exclusion criteria. Through the full text analysis, 172 additional studies were rejected. Our systematic review thus identified 54 unique studies meeting inclusion criteria and further assessed for data extraction. Complete results of the search and review process are detailed in the PRISMA flow diagram (Fig 1).

### General study characteristics

Of the 54 included studies in our review, twenty-seven (50%) were randomized trials (Table 1). The remaining twenty-seven studies (50%) were quasi-experimental studies which used some variation of pre-post test design to analyze data at baseline and post-intervention (Table 2). Studies varied widely. The interventions ranged from 5 weeks to 5 years in duration. Sample sizes ranged from 23 to >10,000 study participants. Forty of the 54 studies (74%) demonstrated statistically significant improvements in one or more LS7 metrics at the final endpoint of the study.

**Table 1:** Summary of randomized controlled trials.

Randomized trials (n = 27)					
First author (year)	Description of sample used in analysis	Mean age (years)	LS7 metric(s)	Intervention	Results
Ahluwalia (2007)*	173 participants from 20 housing developments; 63% female and 86.9% AA in fruit & vegetable (FV) arm; 83% female and 75.8% AA in smoking cessation arm.	48 in FV arm; 43 in smoking cessation arm	Diet	FV consumption arm: a bag of fresh FV, dietary educational materials; two videos on FV. Smoking cessation arm: 8-week supply of nicotine gum, instructions for use, and educational materials related to quitting. Both arms received five sessions of motivational interviewing.	At week 8 and month 6, FV group consumed 1.58 (p = 0.001) and 0.78 (p = 0.04), respectively, more servings than the cessation group in the past 7 days. The FV arm consumed 3.61 (p = 0.01) and 3.93 (p = 0.01) more FV servings at 8 weeks and 6 months, respectively, in the past 30 days compared to cessation group.
Allicock (2013)*	6 AA churches; 302 participants age 50 or older; 71% female	__	Diet	Body and Soul Program: pastoral support, sampling/preparing fruits and vegetables, healthy foods at church events, motivational interviewing sessions)	0.35 servings/day increase from baseline (3.9 servings/day) in FV consumption (p = 0.04). Study found low reach and suboptimal implementation of program.
Andrews (2007)*	103 AA from 2 subsidized housing developments in Augusta, Georgia	44.5 in intervention group; 33 in the comparison group	Smoking status	Smoking Cessation intervention “Sister to Sister”: nurse-delivered behavioral/empowerment counseling in group format, nicotine replacement therapy (NRT), community health worker personal support. Control: self-help materials and 4 total nurse-led group educational sessions.	Six-month continuous abstinence proportions were 27.5% in intervention group and 5.7% in the control group. The Intervention group had significantly higher social support, self-efficacy, and spiritual wellbeing compared to the control group
Boltri (2011)*	5 churches in rural Georgia; 37 AA participants; 62.2% female	57.2	Blood glucose; BMI	Group-based diabetes prevention program (DPP) lasting either 6 weeks or 16 weeks: weekly sessions led by trained leaders included prayer, discussion, and questions. Sessions adapted from the NIH-DPP curriculum.	Overall changes post-intervention to 12 month follow up: fasting glucose decreased from 108.1 to 101.7 mg/dL (p = 0.037); BMI decreased from 33.2 to 32.6 kg/m2 (p<0.05). No significant difference in outcomes between 6 week and 16 week interventions, but lower implementation costs for 6 week intervention program.
**Froelicher (2010)**	60 AA residents from the Bayview-Hunters Point neighborhood in San Francisco, CA; 71.7% female	46.62	Smoking status	5-week smoking cessation program (control group [CG]) or CG plus community co-developed tobacco industry and media messages (IAM).	11.5% of the CG and 13.6% of the IAM group were smoke-free at 6 months and 5.3% of the CG group and 15.8% of the IAM group remained smoke-free at 12 months. None of the smoking cessation outcomes were significantly different.
Haire-Joshu (2003)*	738 AA parents in the Parent as Teacher (PAT) programs located within 12 school districts in St. Louis, MO; 98% female.	29	Diet	Two phase (Fall to Spring) Culturally-appropriate, dietary change program including personal visits, newsletters and group meetings that are led by parent educators for parents of children from birth to age 3.	There was a net difference of 0.53 servings of fruits and vegetables in baseline and post-testing between intervention and control groups (p = 0.03), no significant difference in percent calories from fat consumed daily, and no significant difference in parental modeling behaviors to their children. Parents who received at least one personal visit showed significant reductions in fat intake (p = 0.02) and improvements in low fat eating behaviors (p = 0.01), but no significant improvements in modeling or fruit and vegetable intake.
**Harmon 2014**	23 AA participants; 69.6% female.	62 (intervention arm); 60 control arm	Diet	12-weekly experiential healthy cooking classes followed by 4 monthly booster sessions over 8 months.	There were no significant differences in fruit and vegetable consumption, fat intake, and body weight between the intervention and control groups over the 8 month study period.
**Kegler (2016)**	349 overweight or obese women in rural Georgia; 84.8% AA females.	50.2	Physical activity	16-week tailored home environmental profile (with 3 home visits and 4 coaching calls), goal setting and behavioral contracting for 6 healthy actions. The control group received 3 mailings of educational materials at 6-week intervals.	There were no statistically significant changes in moderate to vigorous physical activity. Intervention participants had significantly higher self-reported weight loss (mean = -9.1 pounds) at 6 months than control participants (p = 0.03). The difference in weight loss was not significant at 12 months, but longitudinal intention-to-treat analyses demonstrated significant differences in weight loss over time (p = 0.03).
Keller (2004)*	29 obese AA females	54.53	Blood cholesterol	36 week low intensity walking program; Group 1 walked 3 days per week and group 2 walked 5 days per week	Walking for 30 minutes for 3 or 5 days a week resulted in clinical increases in serum HDL-C and statistically significant reductions of body fat estimated by bioelectrical impedance analysis and regional fat estimated by waist-to-hip ratio.
**Leone (2016)**	19 AA churches; 712 participants; 68.6% female.	62.8	Physical activity	Colon cancer screening intervention participants (10 churches) received 3 mailed tailored newsletters addressing colorectal cancer screening and physical activity behaviors over approximately 6 months. Nine comparison churches received *Body & Soul*, a fruit and vegetable promotion program.	At follow-up, there were no significant differences overall (p = 0.15) in changes in moderate-vigorous physical activity for intervention versus the comparison group. Reported screening rates were higher at follow-up for both groups, but there were no statistically significant differences between intervention and comparison groups (p = 0.37).
**Levine (2003)**	789 AA residents in the Sandtown-Winchester community of Baltimore, MD; 62% female.	54	Blood pressure	40-month interventions: Less intense arm (usual care, community hypertension [HTN] education, HTN patient education materials) vs more intense arm (less intense intervention + a series of 5 home visits by trained community health workers over a 30 month period including education and counseling, outreach and follow-up and social support mobilization).	There was a mean systolic blood pressure (BP) change of 2.7 mmHg and mean diastolic BP change of 3 mmHg in the more intense arm and mean changes of 6.5mmHg ad 4.6 mmHg, respectively, in the less intense arm. Differences from baseline to follow-up were significant in both arms (p = 0.05). The difference at final follow-up was not (p = 0.10). The percentage of individuals with normal BP increased by 12% and 14% in the more and less intense groups, respectively.
**Miller (2016)**	123 AA; 71% female.	58.6	Blood pressure; Diet; blood cholesterol; blood glucose	Intervention group: coach-directed, tailored DASH diet advice via a 1-hour, in-person, one-on-one session with the study coach who delivered a dietitian-developed module on adoption of the DASH diet and weekly 15-minute calls and money to shop for pre-selected food items over the 8 weeks). Control group: printed materials on improving BP control by adoption of the DASH diet at 15-minute visit with the study coordinator along with money to shop for food throughout the 8 weeks	There was no significant difference in change in BP between the DASH-Plus compared to the control group. There were significant increases in potassium, magnesium, vitamin C, and fiber in the DASH-Plus group compared with the control group (p<0.04), but no change in self-reported consumption of daily dietary fat or cholesterol. The DASH-plus intervention had a significant effect on glucose control among those with diabetes at baseline (p = 0.002)
Morisky (1982)*	200 hypertensive patients from an inner city. 91% AA and 70% female	__	Blood pressure	Family support intervention consisted of a home visit with a lay community person who conducted education concerning hypertension and its management, followed by education using a family member booklet. Small group sessions were the second intervention designed to increase the patient’s understanding and feeling of control over the medical regimen. Group 1 only had family support intervention. Group 2 had family support intervention and small group sessions.	62% of those in Group 2 and 46% in Group 1 had significant difference in final blood pressure control status (p < 0.05) after the educational program.
**Resnick (2008)**	166 participants; 72% AA; 81% female.	73	Physical activity	12- week combined physical activity (stretching, resistance and aerobic exercises according to *Exercise*: *A Guide from the National Institute on Aging*) and efficacy-enhancing (once a week for 30 minutes) intervention. The control group received nutrition education twice weekly for 1–1.5 hours in a group setting.	The intervention group had significantly higher outcome expectations related to exercise (p = .02) and spent more time in exercise (p = .04) than those in the control group. There were no significant differences in self-efficacy expectations (p = .21) or overall physical activity (p = .63) between the two groups.
Resnicow (2009)*	468 AA participants; 73% female.	49	Diet	Three-month intervention: three print-based fruit and vegetable (F & V) newsletters based on individual ethnic identity (EI) over three months. Control: three print-based F & V newsletters tailored only on demographic, behavioral, and social cognitive variables.	Intervention group participants with an Afrocentric EI showed a 1.4 servings per day increase compared to .43 servings among controls (p<0.05). Conversely, there were no between-group differences among those not classified as Afrocentric (n = 338). And, among individuals with a strong EI match, experimental group members increased 1.3 servings compared to .71 among controls (p = .07). For those with weak EI matches, the changes were identical, .89 and .87, respectively, between the two study groups.
**Scarinci (2014)**	565 AA female participants from 6 counties; 495 (88%) participants completed all five sessions.	53.9	Diet; physical activity	The intervention arm consisted of a 5-week healthy lifestyle intervention (four group sessions and one individual session) that was adapted from the “New Leaf… Choices for Healthy Living with Diabetes”. The comparison arm consisted of educational and behavioral strategies to promote breast and cervical cancer screening (four group sessions and one individual session). Both were delivered by lay health workers.	There was a significant change in physical activity between arms (p = 0.004), but the change in physical activity was not associated with any other factors. There was not a significant change in fruit and vegetable consumption. A large percentage of participants in the healthy lifestyle arm reported engaging in physical activity five or more days per week at 12 months (24% increase) as compared to the screening arm (3% increase) (p < .0001). However, physical activity decreased by 16% from baseline at 24 months in the healthy lifestyle group while the screening group increased by 11% from baseline (p = .024).
Schulz (2015)*	695 participants enrolled; 61% AA; 90% female.	46.6	Physical activity; blood pressure; blood glucose; cholesterol; BMI	32- week Community-Health Promoter (CHP)-facilitated walking group intervention. Groups met three times per week at community-based or faith-based organizations, and walked for 45–90 minutes (increasing over time). The control group had a lagged intervention that started at least 8 weeks after the intervention group.	Overall Steps increased significantly with both the number of sessions (p<0.001) and consistency (number of weeks attended at least one session) (p<0.001). Those in the intervention group increased average steps/day about 2000 steps more (p < .0001) compared to those in the lagged group during the same 8-week period. Each increase of 1000 overall steps at 8 weeks compared to baseline was associated with lower odds of high blood pressure (p = 0.01), and with reductions in total cholesterol (p = 0.02), high density lipoprotein (p = 0.00), blood glucose (p = 0.04) and waist circumference (p = 0.01). BMI (p = 0.04).
**Skolarus (2018)**	94 participants; 97% AA; 79% female.	58	Blood pressure	Reach Out, a faith-collaborative, mobile health intervention, consisted of four components: prompted BP self-monitoring, tailored text messages related to these BP results, targeted healthy behavior text messages, and generic healthy behavior text messages. There was a 1:1 ratio intervention to control group allocation.	There were no between-group differences in the change from pre- to post intervention systolic or diastolic blood pressure.
Spencer (2011)*	164 AA and Latino adult participants; Intervention group: 53% AA and 75% female; Control group: 61% AA and 67% female.	50 in intervention group; 55 in control group	Blood glucose (via Hemoglobin A1c)	During the 6-month intervention period, trained community health workers delivered culturally tailored group diabetes education classes in both English and Spanish, conducted two home visits to address participants’ specific self-management goals, and went to 1 clinic visit with the participant and his or her primary care provider. They also contacted intervention participants by phone every 2 weeks. Participants in the control group were contacted once per month to update contact information.	Intervention group participants had a mean HbA1c value improvement of -0.8 percentage points from 8.6% at baseline, to 7.8% at 6 months (P < .01). There was no change in mean HbA1c among the control group. Intervention participants also had significantly greater improvements in self-reported diabetes understanding compared with the control group.
**Tan (2006)**	113 participants; 96% AA; 94% female	69	Physical activity	Experience Corp (EC) Participation: high-intensity volunteering program (15 or more hours per week in public elementary schools) over 8 months.	At 4–8 months, EC participants reported increase in mean minutes expended/week in PA, from 220 to 270 min/week. Control Group decreased from 170 to 140 minutes. This was not significant and the study was not powered to detect a difference. EC participants had 40% increase in Kcal expended compared to a decrease of 16% in controls (not significant). At follow-up, 40% of EC participants met Healthy People 2020 goals. Compared to 25% of controls (p = 0.46).
Tucker (2017)*	70 overweight/obese AA participants; 81.4% female.	__	Diet; physical activity; blood pressure; BMI	6-week church-based health empowerment program designed to promote healthy behaviors. A goal-setting session during week 1 and was followed by four weekly, 90-min meeting sessions that were led by two of the health empowerment coaches. Components included individualized coaching, family health self-empowerment, health-smart behavior resource guide, individual and group exercise (150 mins/week), and provider and community member health panel.	The intervention group, as compared to a waitlist control group, demonstrated a significant increase in the level of healthy eating (p = .001) and physical activity (p < .001). There was no significant change in blood pressure or BMI.
Voorhees (1996)*	22 AA churches; 69% female	46.1 in intensive intervention group; 47.05 in minimal intervention group	Smoking status	Intensive culturally specific, spiritually-based, intervention incorporated smoking behavioral stages of change versus minimal self-help intervention also administered in churches. Analysis compared the two intervention groups at 1-year follow-up with baseline stages.	Differences between interventions for self-reported and biochemically validated quit rates were not statistically significant, but both the intensive (p < 0.0001) and minimal (p < 0.0001) intervention groups differed significantly from the spontaneous quit rates reported in churchgoers in the community reference population. The intensive intervention group was more likely to make positive progress along the stages of change continuum, compared with the self-help intervention group (OR = 1.68; P = 0.04).
Wilcox (2007)*	20 AA churches; 571 participants; 68% female.	__	Physical activity	Faith-based and volunteer-led program with physical activity messaging, teaching behavior change skills for physical activity and healthy eating, use of a 10 min exercise CD, and action-oriented programs including praise aerobics, chair exercises and walking programs.	There was no significant change in moderate-intensity physical activity, meeting physical activity recommendations, or physical activity stage of readiness.
**Wilcox (2013)**	Seventy-four African Methodist Episcopal (AME) churches in South Carolina and 1257 members within them participated in the study; 99.4% AA; 76% female.	54.1	Physical activity; Diet; blood pressure	15-month physical activity and dietary intervention (immediate vs delayed [control]) that targeted social, cultural and policy influences within the African Methodist Episcopal churches. Churches were asked to distribute bulletin inserts (provided); share messages from the pulpit; pass out educational materials (provided); create a Faith, Activity, and Nutrition (FAN) Program bulletin board, and suggest physical activity and healthy eating policy/practices that the pastor could set.	There was a significant effect favoring the intervention group in self-reported leisure- time moderate-to-vigorous physical activity (MVPA) (*p* = 0.02), but no effect for other outcomes.
**Wilson (2015)**	434 AA; 62% female.	51	Physical activity	24-month intervention: Three matched communities were randomized to a police-patrolled walking plus social marketing, a police- patrolled walking-only, or a no-walking intervention.	There were no significant differences across communities for moderate-to-vigorous physical activity.
Woods (2013)*	Five churches; 106 participants; 90% AA; 73% female.	__	BMI; blood pressure; physical activity	Intervention group: Live Well By Faith 8-week program included nutrition as well as 30-minute physical activity components and individualized wellness plans. Minimal intervention control group: Single 90-minute educational workshop at church where basic information was provided about diet, nutrition, exercise and cancer screening. This also included an exercise demonstration of the home-based program, and distribution of the same print materials, pedometer and resistance band given to the intervention group.	At 2-months follow up, the intervention group, compared to the control group, showed significant decreases in weight (p < .02), BMI (*p* < .05), and % body fat (p < .03), with a significant increase in physical fitness (*p* < .02). Systolic blood pressure also showed group differences in the predicted direction (p = .10).
Zoellner (2013)*	91 participants; 62% AA; 91% female.	__	Physical activity; BMI	15-week randomized controlled interventions: group 1 was offered fitness sessions and education in healthful eating and physical activity; group 2 was offered fitness sessions only.	Group 1 experienced significantly greater improvements in body mass index (p< .001) and waist circumference (p = .01), compared with group 2. Both groups significantly increased weekly minutes of moderate physical activity (p < .003). Participants in group 1 also had significantly greater weight loss with higher attendance at the education (p < .001) and fitness sessions (p < .001).

*statistically significant outcomes in at least one measure used to assess LS7 metric(s).

**Table 2:** Summary of studies with pre-post design.

Pre-Post test study design (n = 27)					
First author (year)	Description of sample used in analysis	Mean age (years)	LS7 metric(s)	Intervention	Results
Baker (2016)*	397 AA women and men in southeast Missouri	41.7	Blood pressure; BMI	“Men on the Move: Growing Communities (MOTMGC) “program provided nutrition education and expanded access to healthy food choices through 6 community gardens. The intervention also included the DASH+sodium diet, and distribution of produce to local stores and restaurants.	Compared to the control county, participants from the intervention county had statistically significant decline in BMI, reported consuming more fruits and vegetables, and used less sodium and fat in cooking. In the intervention county, there was a 4 mmHg reduction in systolic blood pressure and 2 mmHg in diastolic blood pressure compared to baseline.
Brewer (2019)*	50 AA from 5 Minnesota churches, 70% female	49.6	All	Participants engaged in a 10-week program using the Fostering African-American Improvement in Total Health [FAITH!] digital application. The mobile app was used for educational modules on cardiovascular health, interactive self-monitoring of diet and exercise behaviors, and a social networking discussion board to interact and share progress with other users.	There were statistically significant reductions in systolic blood pressure (-6.2 mmHg, p = 0.002) and diastolic blood pressure (-5.7 mmHg, p<0.001). There was also an increase in the number of fruit/vegetable servings per day (+1.1 servings/day, p<0.001) as well as the minutes per week of moderate intensity physical activity (+40 mins/week, p = 0.04).
**Brewer (2017)**	37 adult congregants from 3 churches in Rochester, Minnesota, 70% women	51.7	All	16 week educational series implementing Life’s Simple 7 (LS7) framework, adapted from “FAITH” nutrition program for lifestyle modification to decrease CVD risk. Participants received educational manuals and healthy cookbooks, as well as eight 90-minute biweekly sessions which included prayers, personal reflection, lectures, cooking and exercise activities.	There were statistically significant improvements in cardiovascular health status with an increased percent of participants meeting ideal or intermediate LS7 scores from 70% at baseline to 82% at 3 months post-intervention. Higher scores correlated with higher psychosocial measures ratings which assessed socioeconomic status, life outlook, self-reported health, self-efficacy, and family support. Also, cardiovascular health knowledge scores increased from 48% at baseline to 57% post-intervention.
Brown (2017)*	28 adult AA women from churches in Boston, MA. Participants had to be sedentary with BMI greater than 25.	50.5	Physical activity; diet; blood pressure; weight	6 month intervention with Change Club civic engagement curriculum. Weekly meetings with months 1–3 focused on planning and goal setting and months 3–6 focused on implementing action plans related to chosen community improvement project. Monitoring of weight, physical activity, dietary intake, cardiovascular fitness (VO2 max on 1 mile walk test), and BP throughout the program.	Participants met action steps and goals for chosen community projects. Pre and post civic engagement intervention changes included statistically significant improvement in finish time by 1.89 min for 1 mile cardiorespiratory fitness test and decrease in systolic blood pressure by 12.73 mmHg (p<0.001). No statistically significant improvements in other measures: dietary outcomes, physical activity, weight, waist circumference, and body fat percentage.
**Cené (2013)**	104 AA adults from 3 rural counties in North Carolina; participants had high risk for diabetes or self-reported diabetes	57	Blood pressure; blood glucose; BMI	“Power to Prevent Diabetes” curriculum: 12 sessions led by community health ambassadors which spanned 7.5 months and focused on behavior changes for diabetes control as well as food and activity tracking.	Diabetes knowledge increased from 64% at baseline to 80% at 6 months. Self reported physical activity in past week also increased from baseline. There were no statistically significant changes in goals and expectations about diabetes prevention, blood pressure, random blood glucose, or BMI from baseline to 6 months.
Darity (2007)*	2544 AA adult smokers	Not provided	Smoking status	Active intervention: community organizing strategies, direct interpersonal educational activities, and mass media. Passive intervention: mass media only (control group). Cross sectional surveys collected at baseline as well as 6 months, 12 months, and 18 months post intervention.	At 18 months follow up, the point prevalence rate of non-smoking in the active intervention group was 16.7% and 11.8% in the passive group (p = 0.012). The period prevalence of attempting to quite at least once was 33.8% in the active intervention group and 26.2% in the passive intervention group.
**DeMarco (2016)**	AA from a rural North Carolina church community: 17 youth (38% female, 65% healthy weight) and 23 adults (55% female, 74% obese)	Youth: 14.4; Adults: 53.5	Diet; BMI; Blood pressure	12-month church garden nutrition program with 36 total weekly study workshops led by community research director focused on hands-on gardening, nutrition education, and healthy recipe taste-testing.	There was limited impact on nutrition knowledge and behaviors with only youth having statistically significant improvement in gardening and farming attitudes. No statistically significant improvements in FV consumption, BMI, or blood pressure in youth or adults. Higher attendance was positively correlated with a reduction in BP in adults and positive gardening attitudes in youth.
Dodani (2010)*	40 AA church members in Augusta, Georgia with BMI ≥ 25 at high-risk for diabetes. 85% female	46	Weight	The “Fit Body and Soul” group lifestyle intervention was modified from Diabetes Prevention Program and included 12 one-hour, spiritually-based sessions. The program was delivered by church health advisors and pastors.	87.5% of participants attended 10 out of 12 sessions, of whom 88% lost more than 5% of baseline bodyweight.
Dulin-Keita (2015)*	184 AA adults from a public housing community in Birmingham, Alabama, 48% women.	45	Physical activity	HOPE VI (Housing Opportunities for People Everywhere) intervention aimed at public housing neighborhood revitalization to promote leisure physical activity by increasing neighborhood green spaces, play areas, walkways, bikepaths, and improving safety.	There was 77% retention of participants from baseline to 6 months post-HOPE VI. Improved neighborhood walkability increased the odds of neighborhood-based physical activity (P = 0.04). Perceived intervention-related safety improvements increased odds of physical activity by 19% (P = 0.04).
Forthofer (2019)[35]	293 participants from medically underserved communities in Sumter County, South Carolina. 67% AA and 86% female	49.5	Physical activity	*Sumter County on the Move*! Is a 6-month walking intervention. participants formed their own Walking groups consisting of 4–8 members of their existing social networks. The program also included skill-building workshops and participants received a handbook with local community maps and trails for physical activity.	There was significant improvement in goal setting as well as social support for physical activity. At 6 months, there was a decrease in self-reported minutes per day of sitting (p = 0.02) and increase in self-reported moderate physical activity (p = 0.03). However, objective measures of physical activity did not show statistically significant improvements.
Gitlin (2008)*	519 AA elderly participants; 86% female, 59.4% had at least 3 chronic conditions	73	Physical activity	6-week program aimed at management of common symptoms and promotion of healthy behaviors. Peer facilitators taught techniques to improve symptom management through exercise, nutrition, and stress reduction.	There were small but statistically significant improvement in exercise (P = 0.001). Outcomes did not differ by the number of sessions attended or type of chronic conditions.
Goldfinger (2008)*	26 overweight and obese AA church members from Harlem, 81% female	68	BMI; Diet; physical activity	10-week, peer-led, nutrition and physical activity course with key messages of portion control, filling half the plate with fruits and vegetables at each meal, drinking calorie-free beverages, cutting fat, making daily life more active and eating healthy on a budget.	Participants lost an average of 4.4 pounds (P< 0.001) at 10 weeks and 10 pounds at one-year follow-up (P = 0.001). There were also significant reductions in daily fat and an increase in fruit and vegetable intake. Although engagement in exercise did not increase significantly, amount of sedentary time decreased by more than 1 hour per day at 10 weeks (P = 0.034) and by 3 hours at 1 year (P<0.001).
Kim (2008)*	73 rural AA from rural North Carolina, 71% women	54	Weight; Physical activity; Diet	8 week WORD (Wholeness, Oneness, Righteousness, Deliverance) weight loss program which met weekly for 2 hours. Session were led by trained community members and incorporated faith with health education.	The intervention group lost a mean of 3 pounds (P = 0.001) and had a 2.5 cm decrease in hip circumference (P = 0.04) as compared to the control group. Treatment participants reported greater recreational physical activity from baseline to follow-up (P = 0.01). Improvements in diet were not statistically significant.
Liao (2015)*	16 AA communities and 14 comparison communities. The final study samples were 7984 respondents for years 2001–2002, 11,536 for 2002–2003, 11,594 for 2003–2004, 11,772 for 2004–2005, and 10,584 for 2006.	__	Diet	During the 5 year REACH (Racial and Ethnic Approaches to Community Health), there were various health education and promotion programs and social marketing interventions. Community coalitions advocated for policy changes to encourage retailers to sell healthy foods in disadvantaged areas. Neighborhood farmers markets, produce stands and community gardens were set up to increase access to affordable produce including fruits and vegetables.	5-year decrease in fruit juice consumption ranged from −14.3% in REACH 2010 communities to −25.5% in the comparison white population (P<0.001). Daily fruit consumption increased a similar amount in all groups (P <0.01). Vegetable consumption increased in REACH communities (+7.9%; P <0.001), but not in whites (−1.0%; P = 0.059) and blacks (−2.6%; P = 0.136) in the comparison states. Combined fruit and vegetable consumption increased in REACH communities (+7.4%; P <0.001). Little change was observed in the comparison white (+0.6%; P = 0.247) and black population (+0.4%; P = 0.784).
Lynch (2018)*	206 AA church members, 90% female	57.5	Diet; BMI; blood pressure	9-month church-based Abundant Living in Vibrant Energy (ALIVE) intervention consisted of bible study, small group nutrition education sessions, and church-wide activities. Sessions were delivered by trained pastors and other church leaders.	There was an increase from baseline daily vegetable intake by one serving (p < 0.001) as well as weight reduction of 1 kg post-intervention (p < 0.001). There were also significant reductions in blood pressure: systolic blood pressure decreased by 3.91 mmHg (p = 0.002) and diastolic blood pressure decreased by 2.18 mmHg (p = 0.001).
Mitchell (2013)*	48 female participants who were overweight or obese; 98% AA	69.6	BMI	Take Off Pounds Sensibly (TOPS) is a low cost, nationally available, peer-led, nonprofit weight loss program. The 52-week intervention included a one-year membership in TOPS, booklet with a six-week lesson plan, and one-year subscription to TOPS News. They also received a TOPS Wellness Toolkit (a weight management lifestyle guide and workbook, food diary, nutrition guide, achievement log, journal, and resistance bands).	At 52 weeks, 33% of participants lost 5% or more of their initial weight and 48% of participants were clinically weight stable (0–4.9% weight loss) and thus did not experience weight gain.
Mitchell (2018)*[36]	40 rural AA from the Black Belt region of Alabama	__	BMI; physical activity; blood pressure	The Living in Victory Everyday (LIVE) program was a 3 month nutrition and physical activity intervention consisting of group sessions twice per week. Group exercises were tailored to participants’ abilities and new techniques were taught to be performed at home as well. The diet component included educational sessions as well as grocery store tours for shopping on a budget, and healthy food tasting.	There was no statistically significant decreases in BMI and blood pressure post-intervention. Some measures of physical activity improved such as leg balance (p = 0.02) and speed of the 30-second chair stand test (p = 0.02). However, these changes did not impact average weekday sit time.
Parker (2010)*	29 overweight/obese AA women from two rural South Carolinian churches.	51.14	Blood Pressure; BMI; physical activity	10 week weight-loss education program with spiritually-based or nonspiritually-based intervention groups. Both curricula included content on diet, activities, and discussions with health providers. Spiritually-based program also included biblical scriptures.	Nonspiritual group (n = 9) indicated statistically significant reductions in weight (p = 0.05) and systolic blood pressure (p = 0.05). Results for the spiritual group (n = 19) indicated statistically significant reductions in weight (p<0.01), systolic blood pressure (p = 0.05), as well as BMI (p = 0.01) and improvement in physical activity (p<0.01).
Pinsker (2017)*	310 church members from 20 churches. 77% female	__	Diet; physical activity	12-week Body and Soul program, which includes demonstrations of healthy recipes and peer counseling using motivational approaches after services in 20 churches.	The average weekly servings of fruit (p < .001), and vegetables (p < .001) increased from baseline. Level of physical activity on a scale of 1 to 6 in the previous 2 weeks also increased from baseline to follow-up (p = .01).
Plescia (2008)*	908 to 1028 respondents per year over the 5 year period; 95% AA	Not provided	Physical activity; Smoking status; Diet	A multifaceted approach including a community coalition formation lay health advisor (LHA) program and policy and community environment change strategies over a 5-year period. Four main objectives for improving the community environment and public policy included: increasing community resources to remove barriers to healthy behavior, improving quality of care, initiating campaigns to change social norms, and engaging in political advocacy	Improvements were statistically significant for physical activity (P = .02) and smoking (P = .03) among women and for physical activity among middle-aged adults (P = .01). Lower baseline physical activity rates improved to levels comparable to those of African Americans statewide (2001, P < .001; 2005, P = .38), and comparable fruit and vegetable consumption rates became significantly higher (2001, P = .68; 2005, P < .001).
Rodriguez (2012)*	34 AA women with high rates of hypertension (79%), obesity (79%), and elevated waist circumference (94%).	48	Blood pressure; BMI	12-week culturally-tailored weight management intervention focused on nutrition and physical activity where participants received free gym memberships, weekly group sessions lasting 2 hours, group discussions, weigh-ins, blood pressure screenings, free heart-healthy meals during each session, and a supermarket visit to model healthy and affordable food choices.	Reduction of 8 mmHg in systolic blood pressure (p < 0.01) and 1.3 kg/m2 in BMI (p < 0.001) from baseline to follow-up. Although the prevalence of obesity was unchanged, women lost an average of 8.2 pounds during the intervention period (p <0.001).
Salihu (2016)*	49 low-income women (95.9% AA) in Tampa, Florida	31	Diet; physical activity; BMI; blood pressure	8-week Fortified Diet Intervention (FDI) consisting dietary, physical activity, and mental health components. Weekly sessions lasted 2 hours and were facilitated by community members and professionals. The control consisted of individualized information, including meal plans, healthy recipes, simple food substitutions, healthy snack and eating out options, and guidelines for healthy food preparations.	Both the physical fitness subscores (p = 0.002) and nutritional subscores (p = 0.001) increased in the FDI group. These subscores consisted of participant responses to a behavior rating instrument. Postintervention, there were no significant improvements in blood pressure or BMI. No significant improvements were noted in the control group for physical fitness or nutrition sub scores, blood pressure, and BMI.
Two Feathers (2005)*	151 AA and Latino adults with diabetes from 3 health care systems in Detroit, Michigan. 64% AA among whom 78% were women.	59	Blood glucose; Diet	The Racial and Ethnic Approaches to Community Health (REACH) Detroit Partnership diabetes lifestyle intervention aimed at improving diabetes self-care by promoting healthy eating, physical activity, and stress reduction. The curriculum was delivered through 5 group meetings every 4 weeks and was taught by trained community members in both English and Spanish.	Participants from the REACH intervention group had improved A1C values compared to baseline (P< 0.0001). There were also statistically significant improvement in diet including increased consumption of vegetables (P = 0.001), whole grains (P = 0.004), and reduced consumption of soda and sugary beverages (P<0.0001).
**Yancey (2006)**	700 staff, members, or clients from 35 non-profit agencies. 77% AA and 84.5% Female.	48.5	Diet; BMI; Physical activity	The 12 or 6 week intervention involved an organization wellness program consisting of fitness instruction, nutrition education, and physical activity promotion during weekly 30-minute training sessions.	Among the 12-week intervention group, fruit and vegetable intake increased significantly by 0.5 servings/day (P = .00), and body mass index decreased by 0.5 kg/m2 (P = .08). The numbers of days in which individuals participated in vigorous physical activity increased significantly among 6-week intervention participants (P = 0.00).
Yeary (2011)*	26 AA participants from 3 churches in Arkansas, 85% Female, Mean BMI 36.	51	Body weight	Community and academic partners adapted the Diabetes Prevention Program for rural AA church members. The 16-week intervention consisted of faith-based lessons with Bible study and group exercise. The weight goal was 7% reduction of initial body weight. The program provided dietary goals and physical activity targets to help promote the weight loss.	Participants lost an average of 2.34 kg after the 16-week intervention when compared to baseline. There were also significantly increased physical activity during the intervention period. Changes in dietary intake were not significant. Among participants in the 16-week program, weight loss was 4.04 kg in the engaged group (median, −3.13.kg; IQR, −6.71 kg to 0.05 kg) compared with 0.29 kg in the less engaged group (median, −0.14 kg; IQR, −1.54 to 0.64 kg).
**Zoellner (2007)**	83 participants from a rural Mississippi Delta community, 99% AA, 97% Female.	__	BMI; physical activity; blood pressure; blood glucose; cholesterol	This 6-month walking intervention led by supportive trained coaches from the community who promoted goal setting and encouraged walking. Five 1-hour sessions were also delivered during the intervention and focused on weight and dietary behaviors.	Of the 83 enrolled participants, 66 (80%) completed the intervention. Participants exhibited significant improvements in systolic blood pressure (-4.3 mmHg), and high-density lipoprotein (HDL) cholesterol (+7.9 mg/clL), (P < .001). There were no significant reductions in BMI. Although there was net increase from baseline in average minutes of self-reported walking, these changes were not significant.
Zoellner (2014)*	269 participants from Hattiesburg, Mississippi. 94% AA and 85% female.	44	Blood pressure; Diet	6-month intervention included motivational enhancement, social support from peer coaches, pedometer diary self-monitoring, and 90 minute monthly educational sessions led by trained community health professionals focused on the Dietary Approaches to Stop Hypertension (DASH) diet and physical activity.	After the 6 month lifestyle intervention, systolic BP (P = 0.0002) and diastolic BP (P<0.0001) were significantly reduced compared to baseline. Sugar intake also decreased significantly (P<0.0001).

*statistically significant outcomes in at least one measure used to assess LS7 metric(s).

### Formative research

Formative research was employed in 36 studies (67%) to guide intervention development and implementation. The majority of these (72%) achieved statistically significant outcomes in the intended LS7 target(s). Information on community needs, perceptions, and values were primarily gathered during planning phases *before* an intervention began, often with input from community-partners or stakeholders. Some studies also incorporated formative assessments *during* or *after* the intervention in order to evaluate program appropriateness and participant satisfaction. Various methods were identified: interviews were used in 8 studies, focus groups were used in 16 studies, and qualitative or quantitative surveys were used in 14 studies. Less commonly, other formative research approaches such as direct observation were also used [37–40].

### Study quality

With regards to the quality assessment, 11 studies (20%) were rated as good; 35 studies (65%) were rated as fair; and 8 studies (15%) were rated as poor. Among controlled studies (i.e. randomized trials), the most common limitations to quality were lack of double blinding as well as drop-out rates > 20% at the study end-point. In many studies, authors also failed to report that the sample size was adequately large to detect outcome differences with sufficient power. Among pre-post test studies, many lacked the following features: enrollment of all eligible participants who met inclusion criteria, a sufficiently large sample size to provide confidence, blinding of study evaluators to the participants’ intervention exposures, and use of interrupted time-series design. Notably, compared to pre-post test and other study designs, randomized trials were more likely to have an overall quality rating of good to fair.

### Target population

No studies were specifically designed for African American men. Seven studies specifically targeted African American women [41–47]. Of the remaining 47 studies aimed at both African American men and women, 32 studies had females accounting for more than 70% of participants.

### Setting

All studies were conducted in the community setting. Specifically, 22 (41%) of the selected studies described faith-based programs. Two studies included schools [48, 49] and 3 took place in public housing developments [44, 50, 51].

### Study outcomes

The intervention outcomes of 27 studies included physical activity; 21 included blood pressure; 23 included weight or BMI; 7 included smoking status; 9 included blood glucose; 6 included cholesterol; and 23 included change in diet.

Only two studies evaluated changes in all LS7 components [52, 53]. These interventions were related to the faith-based nutrition and exercise program conducted in AA churches in Minnesota: “Fostering African-American Improvement in Total Health [FAITH!].” [52, 53] Overall, twenty-seven (50%) studies targeted more than one LS7 metric. Such interventions, with > 1 LS7 metric, were more likely than not (70%) to have statistically significant improvements in outcome measures. For studies with ≥3 LS7 metrics, the most commonly combined targets were blood pressure, physical activity, and BMI/weight. Diet interventions were most likely to be paired with physical activity. Interventions targeting blood glucose most often incorporated blood pressure and/or BMI/weight components.

#### Physical activity

Sixteen out of 27 studies measuring physical activity achieved statistically significant increases in levels of physical activity post-intervention. Data primarily consisted of participants’ self-reported behaviors gathered through surveys and activity ranking scales which varied widely between studies. Other measures included number of steps per day and weekly time expenditures engaging in recreational or moderate to vigorous physical activity. Among effective interventions, almost all delivered educational materials focused on promoting greater at-home activity levels and developing weekly routines to meet fitness goals. Of the effective interventions, a majority created fitness goals and other physical activity plans with groups facilitated by coaches or leaders recruited from the community to better represent and serve said population [54–59]. Other studies allowed participants to establish individualized goals and activity plans [60, 61]. In addition, studies also incorporated supervised exercise ranging from 10 to 90 minutes per week during group sessions. Such activities included walking, stretching, aerobics, strength training, and dancing [35, 38, 39, 61–63]. Few interventions targeted local environments through community and policy changes to encourage neighborhood walking/jogging as well as recreational physical activity by increasing bike paths and play areas [51, 64]. Overall, studies that utilized multiple approaches to encourage activity (such as group exercise in addition to tailored curriculum or at-home activities) were more effective than those using only one form of intervention.

#### Blood pressure

Only 10 out of the 21 studies which measured changes in blood pressure achieved statistically significant blood pressure reduction. Among these studies, systolic blood pressure decreased by 8.17 mmHg on average following intervention [43, 45, 53, 65–68]. Physical activity and diet change were the main approaches for attaining blood pressure control. Coach led walking groups were commonly employed methods [39, 45, 65, 66]. Nutritional components emphasized the DASH diet (Dietary Approaches to Stop Hypertension) in conjunction with the use of community gardens and assistance with healthy food shopping [43, 45, 65, 66, 69]. Interventions ranged in duration from 6 weeks to 5 years; however, longer duration did not consistently correlate with the effectiveness of the intervention. Faith based initiatives used similar intervention techniques but had a lower percentage of studies achieving statistically significant outcomes with regards to blood pressure management [43, 60, 61, 70, 71].

#### BMI

Thirteen of the 23 studies evaluating body weight or BMI achieved statistically significant results. Almost all studies promoted wellness plans targeting diet and physical activity to meet weight loss goals. This was done primarily in the group setting, such as group exercise sessions; however, few studies incorporated individualized meetings to discuss weight management. Three interventions used curricula which were guided by the Diabetes Prevention Program (DPP) [40, 72, 73]. Effective interventions varied in duration and ranged from 6 weeks to 5 years. Among these, many employed faith-based programs or recruited participants from church communities [39, 40, 43, 52, 60, 63, 68, 72, 74]. The average reduction in BMI was 2.03 kg/m^2^ [40, 43, 45, 72, 75]. Average weight loss was 4.36 pounds [40, 43, 45, 60, 63, 68, 74, 75]. Other studies reported percent of participants losing greater than 5% of baseline weight or a decrease in the prevalence of those who were overweight or obese [39, 47, 69, 73]. Interventions focused on both physical activity and diet were more likely to achieve statistically significant weight loss than those targeting diet alone.

#### Smoking

Of the seven studies evaluating smoking cessation, four had statistically significant results. Average post-intervention self-reported quit rate was 24% [44, 76, 77]. Studies shared similar intervention methods. Control groups primarily received self-help material such as pamphlets. Mass media campaigns delivering print and electronic communication, including mediums like social media and news/radio stations, were commonly used to promote changes in smoking behavior [64, 77, 78]. In the church setting, scriptural messaging was the primary tool used to encourage smoking cessation [52, 76]. Interventions which incorporated free nicotine replacement therapy generally sustained greater rates of abstinence at long term follow-up when comparing intervention and control groups [44, 78].

#### Glycemia

Six out of the nine studies reporting results in glycemic control evaluated improvements in diabetes as a primary outcome [52, 65, 72, 79–81]. Among the 5 studies with statistically significant results, there was an average decrease in blood glucose of 6.4 mg/dL and Hemoglobin A1c level of 0.7% post-intervention [39, 65, 72, 80, 81]. Interventions commonly implemented lifestyle modifications and used trained coaches to deliver culturally-tailored curricula focused on diet, self-care, and diabetes knowledge [79–82]. Other studies implemented group physical activity and walking programs to attain improved glycemic measures [39, 65]. The intervention duration, which varied from 8 to 32 weeks, did not consistently correlate to the significance of results.

#### Cholesterol

Only six studies evaluated changes in cholesterol. Three of these interventions, which achieved statistically significant results, were longer in duration (31 weeks on average). Although interventions targeted dietary changes, programs primarily focused on physical activity through walking groups as a means to improve blood lipid levels [39, 41, 65, 82]. Among these studies, HDL levels increased by an average of 5.7 mg/dL and total cholesterol was reduced by 2.2 mg/dL [39, 41, 65].

#### Diet

Sixteen of 23 studies reporting dietary outcomes achieved statistically significant results. Diet modifications aimed at increasing fruit and vegetable (FV) intake, whole grains, and fiber, while decreasing consumption of sugary drinks and fat. Diet education also emphasized portion control and healthy snacking habits. Some curricula adapted material from specific diets, such as the DASH diet [66, 82]. Among studies reporting change in fruit and vegetable consumption, there was an average increase in FV intake by 0.7 servings/day [48, 50, 53, 68, 83–86]. In addition to group informational sessions, effective studies also employed hands-on methods such as cooking classes, demonstrations, and taste testing to promote better nutrition [46, 68, 83, 87]. Policy and community changes such as improving access to farmer’s markets, community gardens, and healthier options at local retailers were also effective strategies [64, 88]. Few studies also directly provided participants with produce or money to purchase fresh foods [50, 82]. Intervention duration did not consistently correlate with improved outcomes.

## Discussion

Life’s Simple 7 (LS7) metrics—an AHA framework for ideal cardiovascular health against which reduction in risk for CVD, diabetes, and cancer can be measured—are empirically supported as a means to reduce development of disease and premature mortality. This review provides a comprehensive account of community-based participatory research and community-engaged interventions applying LS7 metrics to AA. Community-engaged interventions can provide more holistic strategies to address upstream factors such as racism and the social determinants of health underlying the mechanisms leading to racial health disparities.

Overall, we found a lack of consensus on best practices to apply and attain the metrics within interventions represented by wide variation in intervention strategies, intervention efficacy, and insufficient study quality to generalize findings. Moreover, quality did not consistently correlate to the statistical significance of study outcomes.

However, we found that implementation of the interventions shared commonalities. In this review, interventions guided by formative research data were found to increase the effectiveness of community programs. Importantly, formative research can aid researchers in both formulating and modifying interventions to be culturally relative and optimize intended effects. Furthermore, faith-based entities and community health workers are common vehicles for health promotion activities and interventions. Faith-based interventions among AAs are heralded as an approach to assist communities with building capacity with other communities [89, 90]. And although there is a need to enhance the training of and educational programs for church and health ministry leaders who deliver such interventions [91], faith-based organizations have a longstanding history of credible health promotion for the AA community and, as such, faith-based partnerships have been recommended as a necessary driver of national health promotion efforts [92, 93]. This is not surprising, given that evidence suggests that more AA engage with a faith-based organization and AA have the highest church attendance rates in the US as compared to other racial and ethnic groups [94–96]. Similarly, utilization of community health workers is an evidence-based approach to navigating AAs beyond barriers to adoption of healthy behaviors and practices (such as mistrust of healthcare providers in medical settings), adherence of provider recommendations, academic-community partnerships and cultivating community capacity for health promotion [97–101]. This review demonstrated statistical benefit of faith and community health worker-based interventions, but the clinical benefit is unclear and generalizability of these findings is significantly limited by the variance in study quality.

Content identified within studies included in this review ranged from one to seven LS7 metrics. The most common targets were diet, weight, and blood pressure, and less than 10% of the reviewed studies addressed smoking status. There is a dire need for comprehensive approaches targeting all seven metrics, as there is a graded relationship between the number of ideal cardiovascular health metrics attained and the risk of cardiovascular disease [12]. This effect is even more pronounced among AA as compared to Whites [11]. Therefore, interventions targeting more than one LS7 metric may have greater potential to substantially reduce black-white disparities in CVD. Further, a more holistic approach to health promotion as opposed to singular targets is both preferred [102] and feasible [52] within AA community. Thus, although inclusion of multiple intervention components and assessments may be difficult to analyze, researchers must become more adept in planning such studies to increase uptake and overall outcomes.

There are limitations to this review. First, this review was conducted in three databases and only included full text, peer-reviewed publications. It is possible that potentially relevant studies to this review were missed due to incomplete retrieval of the existing literature as well as variable indexing across databases. Second, there is a dearth of knowledge surrounding the application of all LS7 metrics within targeted interventions for AAs. Of those that were found with one or more LS7 metrics, most exhibited relatively fair appraisal quality. Primary concerns with quality derived from high attrition rates and lack of power to detect effects. Therefore, findings must be interpreted with caution. There were also variable intervention techniques and metrics used across studies, limiting the ability to identify best practices. More research using randomized experimental designs with sufficient sample sizes and high retention rates is needed to strengthen the literature on application of community-based and–engaged LS7 interventions, especially among AA men who were underrepresented in the current studies.

## Conclusion

This review highlights opportunities for academic-community collaborations in addressing disparities among AAs, gaps in community-engaged and community-based participated research, and various ways to capitalize on community capacity to deliver interventions with some indication that such methods can be acceptable and promising to reduce the CVD burden. Academic institutions engaging the community and establishing shared responsibility can build infrastructure to support the development, implementation and dissemination of acceptable, evidence-based, effective and sustainable interventions for future use [103, 104]. Yet, based on the available studies, there is insufficient data to recommend a specific community-engaged or CBPR intervention to improve attainment of AHA LS7 metrics among AAs. Future studies aiming to reduce disparities in CVD and/or chronic disease such as diabetes and cancer among AAs may benefit from use of the comprehensive AHA LS7 framework, greater engagement of AA males, and be conducted with more rigorous study methodology. High-quality, generalizable studies are of vital importance in identifying strategies to achieve health equity.

## Supporting information

S1 File Result of quality assessment. (XLSX)

S2 File PRISMA checklist LS7 review. PRISMA checklist.
(DOC)

S1 Appendix (DOCX)

## Abbreviations

AA: African American
CVD: Cardiovascular Disease
AHA: American Heart Association
LS7: Life’s Simple 7
CBPR: Community-Based Participatory Research
