# Supplementary material for: Community-engaged and community-based participatory research to promote American Heart Association Life’s Simple 7 among African American adults: A systematic review
Source: PLoS One. 2020 Sep 1;15(9):e0238374. doi: 10.1371/journal.pone.0238374 (PMC7462313; doi:10.1371/journal.pone.0238374)
Supplement: S1 Appendix — (DOCX) [file pone.0238374.s003.docx]

**APPENDIX**

**Search Strategies**

**Pubmed:** (Searched by “most recent”)

(((((((((((cardiovascular diseases [mesh:noexp] OR cardiovascular diseases [tw] OR cardiovascular disease [tw] OR cardiovascular health [tw] OR heart diseases [mesh:noexp] OR heart diseases [tw] OR heart disease [tw])) OR (exercise [mesh:noexp] OR exercise [tw] OR physical activity [tw])) OR (healthy diet [mesh:noexp] OR healthy diet [tw] OR diet [mesh] OR diet [tw] or healthy lifestyle [mesh:noexp] OR healthy lifestyle [tw] OR nutrition [tw])) OR (smoking [mesh] OR smoking [tw] OR smoking prevention [mesh:noexp] OR smoking prevention [tw] OR smoking cessation [mesh:noexp] OR smoking cessation [tw])) OR (body weight [mesh:noexp] OR overweight [mesh:noexp] OR obesity [mesh:noexp] OR body weight [tw] OR overweight [tw] OR obesity [tw] OR weight [tw] OR body mass index [mesh] OR body mass index [tw] OR bmi [tw])) OR (blood pressure [mesh:noexp] OR blood pressure [tw] OR hypertension [mesh:noexp] OR hypertension [tw] or high blood pressure [tw])) OR (blood glucose [mesh:noexp] OR blood glucose [tw] OR blood sugar [tw] OR glycemic control [tw] OR diabetes mellitus [mesh:noexp] OR diabetes [tw] OR glycaemic control [tw])) OR (cholesterol [mesh:noexp] OR cholesterol [tw] OR cholesterol, vldl [mesh:noexp]))) AND (Community-based participatory research [mesh:noexp] OR community-based participatory research [tw] OR cbpr [tw] OR community based research [tw] OR consumer based research [tw] OR consumer driven research [tw] OR community engagement [tw] OR participatory research [tw] OR community participation [mesh:noexp] OR cooperative behavior [mesh:noexp])) AND (African americans [mesh:noexp] OR African americans [tw] OR African American [tw] OR black [tw] OR blacks [tw])

**(#results = 362)**

**Embase:** (Searched by “most broad—with explosion”)

(((((((('cardiovascular'/exp OR cardiovascular) AND ('diseases'/exp OR diseases) OR 'cardiovascular'/exp OR cardiovascular) AND ('diseases'/exp OR diseases) OR 'cardiovascular'/exp OR cardiovascular) AND ('diseases'/exp OR diseases) OR 'cardiovascular'/exp OR cardiovascular) AND ('health'/exp OR health) OR 'heart'/exp OR heart) AND ('diseases'/exp OR diseases) OR 'heart'/exp OR heart) AND ('diseases'/exp OR diseases) OR 'heart'/exp OR heart) AND ('disease'/exp OR disease) OR (('exercise'/exp OR exercise OR physical) AND ('activity'/exp OR activity)) OR ((((healthy AND ('diet'/exp OR diet) OR healthy) AND ('diet'/exp OR diet) OR 'diet'/exp OR diet OR healthy) AND ('lifestyle'/exp OR lifestyle) OR healthy) AND ('lifestyle'/exp OR lifestyle)) OR 'nutrition'/exp OR nutrition OR ((((('smoking'/exp OR smoking) AND ('prevention'/exp OR prevention) OR 'smoking'/exp OR smoking) AND ('prevention'/exp OR prevention) OR 'smoking'/exp OR smoking) AND ('cessation'/exp OR cessation) OR 'smoking'/exp OR smoking) AND ('cessation'/exp OR cessation)) OR ((((('body'/exp OR body) AND ('weight'/exp OR weight) OR 'overweight'/exp OR overweight OR 'obesity'/exp OR obesity OR 'body'/exp OR body) AND ('weight'/exp OR weight) OR 'overweight'/exp OR overweight OR 'obesity'/exp OR obesity OR 'weight'/exp OR weight OR 'body'/exp OR body) AND ('mass'/exp OR mass) AND ('index'/exp OR index) OR 'body'/exp OR body) AND ('mass'/exp OR mass) AND ('index'/exp OR index)) OR 'bmi'/exp OR bmi OR (((('blood'/exp OR blood) AND ('pressure'/exp OR pressure) OR 'blood'/exp OR blood) AND ('pressure'/exp OR pressure) OR 'hypertension'/exp OR hypertension OR high) AND ('blood'/exp OR blood) AND ('pressure'/exp OR pressure)) OR ((((((('blood'/exp OR blood) AND ('glucose'/exp OR glucose) OR 'blood'/exp OR blood) AND ('glucose'/exp OR glucose) OR 'blood'/exp OR blood) AND ('sugar'/exp OR sugar) OR glycemic) AND ('control'/exp OR control) OR 'diabetes'/exp OR diabetes) AND mellitus OR 'diabetes'/exp OR diabetes OR glycaemic) AND ('control'/exp OR control)) OR (('cholesterol'/exp OR cholesterol OR cholesterol,) AND ('vldl'/exp OR vldl))) AND (((((((('community based' AND participatory AND ('research'/exp OR research) OR 'community based') AND participatory AND ('research'/exp OR research) OR cbpr OR 'community'/exp OR community) AND based AND ('research'/exp OR research) OR 'consumer'/exp OR consumer) AND based AND ('research'/exp OR research) OR 'consumer'/exp OR consumer) AND driven AND ('research'/exp OR research) OR 'community'/exp OR community) AND ('engagement'/exp OR engagement) OR participatory) AND ('research'/exp OR research) OR 'community'/exp OR community) AND ('participation'/exp OR participation) OR cooperative) AND ('behavior'/exp OR behavior) AND (((('african'/exp OR african) AND ('americans'/exp OR americans) OR 'african'/exp OR african) AND ('americans'/exp OR americans) OR 'african'/exp OR african) AND ('american'/exp OR american) OR 'black'/exp OR black OR 'blacks'/exp OR blacks)

**(#results = 362)**

**CINAHL:**

( african americans or black americans or blacks ) AND ( community based participatory research or cbpr or action research ) AND ( diabetes mellitus or diabetes or blood glucose or exercise or physical activity or diet or healthy diet or nutrition or smoking cessation or smoking or smoking prevention or overweight or obesity or bmi or body mass index or high blood pressure or hypertension or cholesterol or hyperlipidemia )

**(#results = 242)**

**NIH Study Quality Assessment Tools**

| **Controlled Studies** |
| --- |
| 1. Was the study described as randomized, a randomized trial, a randomized clinical trial, or an RCT? |
| 2. Was the method of randomization adequate (i.e., use of randomly generated assignment)? |
| 3. Was the treatment allocation concealed (so that assignments could not be predicted)? |
| 4. Were study participants and providers blinded to treatment group assignment? |
| 5. Were the people assessing the outcomes blinded to the participants' group assignments? |
| 6. Were the groups similar at baseline on important characteristics that could affect outcomes (e.g., demographics, risk factors, co-morbid conditions)? |
| 7. Was the overall drop-out rate from the study at endpoint 20% or lower of the number allocated to treatment? |
| 8. Was the differential drop-out rate (between treatment groups) at endpoint 15 percentage points or lower? |
| 9. Was there high adherence to the intervention protocols for each treatment group? |
| 10. Were other interventions avoided or similar in the groups (e.g., similar background treatments)? |
| 11. Were outcomes assessed using valid and reliable measures, implemented consistently across all study participants? |
| 12. Did the authors report that the sample size was sufficiently large to be able to detect a difference in the main outcome between groups with at least 80% power? |
| 13. Were outcomes reported or subgroups analyzed prespecified (i.e., identified before analyses were conducted)? |
| 14. Were all randomized participants analyzed in the group to which they were originally assigned, i.e., did they use an intention-to-treat analysis? |

| **Cross-sectional Studies** |
| --- |
| 1. Was the research question or objective in this paper clearly stated? |
| 2. Was the study population clearly specified and defined? |
| 3. Was the participation rate of eligible persons at least 50%? |
| 4. Were all the subjects selected or recruited from the same or similar populations (including the same time period)? Were inclusion and exclusion criteria for being in the study prespecified and applied uniformly to all participants? |
| 5. Was a sample size justification, power description, or variance and effect estimates provided? |
| 6. For the analyses in this paper, were the exposure(s) of interest measured prior to the outcome(s) being measured? |
| 7. Was the timeframe sufficient so that one could reasonably expect to see an association between exposure and outcome if it existed? |
| 8. For exposures that can vary in amount or level, did the study examine different levels of the exposure as related to the outcome (e.g., categories of exposure, or exposure measured as continuous variable)? |
| 9. Were the exposure measures (independent variables) clearly defined, valid, reliable, and implemented consistently across all study participants? |
| 10. Was the exposure(s) assessed more than once over time? |
| 11. Were the outcome measures (dependent variables) clearly defined, valid, reliable, and implemented consistently across all study participants? |
| 12. Were the outcome assessors blinded to the exposure status of participants? |
| 13. Was loss to follow-up after baseline 20% or less? |
| 14. Were key potential confounding variables measured and adjusted statistically for their impact on the relationship between exposure(s) and outcome(s)? |

| **Pre-Post Design (no control)** |
| --- |
| 1. Was the study question or objective clearly stated? |
| 2. Were eligibility/selection criteria for the study population prespecified and clearly described? |
| 3. Were the participants in the study representative of those who would be eligible for the test/service/intervention in the general or clinical population of interest? |
| 4. Were all eligible participants that met the prespecified entry criteria enrolled? |
| 5. Was the sample size sufficiently large to provide confidence in the findings? |
| 6. Was the test/service/intervention clearly described and delivered consistently across the study population? |
| 7. Were the outcome measures prespecified, clearly defined, valid, reliable, and assessed consistently across all study participants? |
| 8. Were the people assessing the outcomes blinded to the participants' exposures/interventions? |
| 9. Was the loss to follow-up after baseline 20% or less? Were those lost to follow-up accounted for in the analysis? |
| 10. Did the statistical methods examine changes in outcome measures from before to after the intervention? Were statistical tests done that provided p values for the pre-to-post changes? |
| 11. Were outcome measures of interest taken multiple times before the intervention and multiple times after the intervention (i.e., did they use an interrupted time-series design)? |
| 12. If the intervention was conducted at a group level (e.g., a whole hospital, a community, etc.) did the statistical analysis take into account the use of individual-level data to determine effects at the group level? |

| **Case-control Studies** |
| --- |
| 1. Was the research question or objective in this paper clearly stated and appropriate? |
| 2. Was the study population clearly specified and defined? |
| 3. Did the authors include a sample size justification? |
| 4. Were controls selected or recruited from the same or similar population that gave rise to the cases (including the same timeframe)? |
| 5. Were the definitions, inclusion and exclusion criteria, algorithms or processes used to identify or select cases and controls valid, reliable, and implemented consistently across all study participants? |
| 6. Were the cases clearly defined and differentiated from controls? |
| 7. If less than 100 percent of eligible cases and/or controls were selected for the study, were the cases and/or controls randomly selected from those eligible? |
| 8. Was there use of concurrent controls? |
| 9. Were the investigators able to confirm that the exposure/risk occurred prior to the development of the condition or event that defined a participant as a case? |
| 10. Were the measures of exposure/risk clearly defined, valid, reliable, and implemented consistently (including the same time period) across all study participants? |
| 11. Were the assessors of exposure/risk blinded to the case or control status of participants? |
| 12. Were key potential confounding variables measured and adjusted statistically in the analyses? If matching was used, did the investigators account for matching during study analysis? |
